# Adsorption of Pyrene and Arsenite by Micro/Nano Carbon Black and Iron Oxide

**DOI:** 10.3390/toxics12040251

**Published:** 2024-03-29

**Authors:** Shuai Zhang, Gulijiazi Yeerkenbieke, Shuai Shi, Zhaoyang Wang, Lijin Yi, Xiaoxia Lu

**Affiliations:** Ministry of Education Laboratory for Earth Surface Processes, College of Urban and Environmental Sciences, Peking University, Beijing 100871, China

**Keywords:** micro/nano carbon black, micro/nano iron oxide, pyrene, As (III), adsorption, desorption

## Abstract

Polycyclic aromatic hydrocarbons (PAHs) and arsenic (As) are common pollutants co-existing in the environment, causing potential hazards to the ecosystem and human health. How their behaviors are affected by micro/nano particles in the environment are still not very clear. Through a series of static adsorption experiments, this study investigated the adsorption of pyrene and arsenite (As (III)) using micro/nano carbon black and iron oxide under different conditions. The objectives were to determine the kinetics and isotherms of the adsorption of pyrene and As (III) using micro/nano carbon black and iron oxide and evaluate the impact of co-existing conditions on the adsorption. The microstructure of micro/nano carbon black (C 94.03%) is spherical-like, with a diameter of 100–200 nm. The micro/nano iron oxide (hematite) has irregular rod-shaped structures, mostly about 1 µm long and 100–200 nm wide. The results show that the micro/nano black carbon easily adsorbed the pyrene, with a pseudo-second-order rate constant of 0.016 mg/(g·h) and an adsorption capacity of 283.23 μg/g at 24 h. The micro/nano iron oxide easily adsorbed As (III), with a pseudo-second-order rate constant of 0.814 mg/(g·h) and an adsorption capacity of 3.45 mg/g at 24 h. The mechanisms of adsorption were mainly chemical reactions. Micro/nano carbon black hardly adsorbed As (III), but its adsorption capability for pyrene was reduced by the presence of As (III), and this effect increased with an increase in the As (III) concentration. The adsorbed pyrene on the micro/nano black carbon could hardly be desorbed. On the other hand, the micro/nano iron oxide could hardly adsorb the pyrene, but its adsorption capability for As (III) was increased by the presence of pyrene, and this effect increased with an increase in the pyrene concentration. The results of this study provide guidance for the risk management and remediation of the environment when there is combined pollution of PAHs and As.

## 1. Introduction

Polycyclic aromatic hydrocarbons (PAHs) and arsenic (As) are common pollutants co-existing in the environment [1,2]. PAHs are mainly produced by human activities such as the incomplete combustion of coal, petroleum, and biomass [1] or by natural activities such as volcanic eruptions and forest fires [3]. Due to their high mutagenicity and carcinogenicity, 16 PAHs have been listed as priority pollutants by the European Union (EU) and the United States Environmental Protection Agency (US EPA) [4]. As is widely present in the natural environment, mainly in the form of arsenic sulfide; however, it also has a wide range of uses in human life, e.g., in industries for alloys, pesticides, pharmaceuticals, and anti-corrosion materials [5], and it can enter environmental media via waste discharge or other approaches [6]. As generally exists in two valence states in soil and water environments: III (+3) and V (+5). As (III) is more harmful than As (V) due to its higher solubility and bioavailability [6]. The long-term intake of food or water with a high As content could cause damage to the human liver, heart, and nervous vessels [7].

Black carbon and iron oxide are common substances in soil and water environments. Black carbon describes a range of carbonaceous substances, from partly charred plant residues to highly graphitized soot, that are generated as products of incomplete combustion [8]; they have variable chemical compositions depending on their sources, sometimes being primarily elemental carbon and sometimes existing as complex mixtures of elemental carbon, organic carbon, and other non-carbon species [9]. Iron oxide is one of the most abundant minerals on Earth’s surface, and it is primarily located in the shallow crust. It is an iron oxide with a chemical composition of Fe_2_O_3_. Micro/nano black carbon and iron oxide are likely present in soil and water environments due to various physical, chemical, and biological processes.

Adsorption is a major process that affects the migration and toxicity of pollutants in the environment. Compared to the corresponding larger particles, micro/nano particles generally have higher adsorption capabilities due to their larger specific surface areas [10,11]. Previous studies have shown that PAHs are easily adsorbed by carbonaceous substances, while As is easily adsorbed by metal oxides [12,13,14,15]. However, how the co-existence of PAHs and As influences their adsorptions is not clear yet. This knowledge gap should be given attention since PAHs and As often co-exist in the environment. 

This study explored the adsorption of pyrene and arsenite (As (III)) using micro/nano carbon black and iron oxide under individual and co-existing conditions through laboratory experiments. The objectives were to determine the kinetics and isotherms of the adsorption of pyrene and As (III) using micro/nano carbon black and iron oxide and evaluate the impact of co-existing conditions on the adsorption. In this study, micro/nano carbon black was used as a surrogate for micro/nano black carbon, which is primarily an elemental carbon. Pyrene and As (III) were used as representatives for PAHs and As, respectively. This is the first time that the impact of the co-existence of PAHs and As on their adsorptions using micro/nano carbon black and iron oxide has been clarified. The results of this study provide a scientific basis for the risk management and remediation of the environment when there is combined pollution of PAHs and As.

## 2. Materials and Methods

### 2.1. Materials

Micro/nano iron oxide (Fe_2_O_3_, purity 99.9%) and sodium arsenite (NaAsO_2_, purity 99%) were purchased from Innochem Science & Technology Co., Ltd. (Beijing, China). Micro/nano carbon black (industrial grade purity) was purchased from Hewns Biochemical Technology Co., Ltd. (Tianjin, China). Pyrene (95% purity) was purchased from Sun Chemical Technology Co, Ltd. (Shanghai, China). Pyrene standard solution (0.2 mg/mL in Dichloromethane) and isotopic-labeled internal standard solutions (Phe-d10 0.2 mg/mL in Dichloromethane and Pyr-d10 1000 μg/mL in Dichloromethane) were purchased from AccuStandard, Inc. (New Haven, CT, USA). Arsenious acid standard solution (18.2 μg/mL) and Arsenic acid standard solution (35.8 μg/mL) were purchased from the National Institute of Metrology (Beijing, China). n-Hexane (HPLC grade) was purchased from Thermo Fisher Scientific Inc. (Waltham, MA, USA).

### 2.2. Characterization of Micro/Nano Particles

Scanning electron microscopy (SEM) was employed to measure the morphology of the studied micro/nano carbon black and iron oxide particles according to the Chinese Standard JY/T 010-1996 [16]. X-ray diffraction (XRD) and Fourier transform infrared spectroscopy (FTIR) were used to study the composition and functional group of iron oxide according to the Chinese Standard SY/T 5163-2018 and GB/T 32199-2015 [17,18]. X-ray photoelectron spectroscopy (XPS) was applied to study the composition of carbon black according to the Chinese Standard JY/T 013-1996 [19].

### 2.3. Adsorption and Desorption Experiments

#### 2.3.1. Pre-Experiment

Pre-experiment of adsorption was performed to determine the optimal ratio of the studied micro/nano particles on pollutants (Table S1). A series of dosages of micro/nano carbon black or iron oxide (0, 5, 10, 25, 50, 100, 500, and 1000 mg) were added to 40 mL glass centrifuge tubes, and then 40 mL of ultrapure water containing 80 μg/L pyrene or 10 mg/L As (III) was added to each tube and shaken in a temperature-controlled shaker (HZQ-2, Jinyi, Changzhou, China) at 200 r/min under 25 °C for 24 h. Thereafter, the tubes were centrifugated in a centrifuge (LD5-10, Jingli, Beijing, China) at 3000 r/min under room temperature for 15 min. The supernatant was taken to measure the concentration of pyrene or As (III). The adsorption capabilities under different dosages were calculated. It turned out that the optimal dosages for micro/nano carbon black and iron oxide were 5 mg and 10 mg, respectively. These dosages were used for further studies.

#### 2.3.2. Adsorption Kinetics

Based on the pre-experiment, micro/nano carbon black easily adsorbed pyrene but hardly adsorb As (III), while micro/nano iron oxide easily adsorbed As (III) but hardly adsorbed pyrene. Therefore, kinetics were performed for the adsorption of pyrene using micro/nano carbon black and the adsorption of As (III) using micro/nano iron oxide. Multiple adsorption tubes were prepared by adding 5 mg micro/nano carbon black and 40 mL pyrene solution (80 μg/L) or 10 mg micro/nano iron oxide and 40 mL As (III) solution (10 mg/L). These tubes were shaken at 200 r/min under 25 °C for different times (1/12, 1/6, 1/4, 1/2, 1, 2, 4, 6, 12, and 24 h). At each time point, three tubes were taken for the analysis of pyrene or As (III) in the supernatant (after centrifuging at 1850× g for 15 min). At 24 h after adsorption, iron oxide was collected for FTIR analysis. In addition, tubes containing 40 mL pyrene solution (80 μg/L) or As (III) (10 mg/L) but no micro/nano carbon black or iron oxide were prepared as controls for losses due to volatilization or other processes.

#### 2.3.3. Adsorption/Desorption Isotherms

For experiments of adsorption isotherms, seven combinations were set up, as shown in Table 1. The concentration of pyrene ranged from 1 to 80 μg/L (1, 2, 5, 10, 20, 40, 60, and 80 µg/L), and the concentration of As (III) ranged from 0.01 to 1 mg/L (0.01, 0.02, 0.05, 0.1, 0.2, 0.4, 0.6, 0.8, and 1 mg/L). All the adsorption tubes were shaken for 24 h (25 °C, 200 r/min), centrifuged for 15 min (1850× g), and analyzed for the target pollutant in the supernatant. The experiments were performed in triplicate.

Experiments of desorption isotherms were performed after the adsorption isotherms experiments were finished. Briefly, the supernatants in the tubes after centrifugation were removed, and equal volumes of ultrapure water were added into the tubes. After being shaken for 24 h (25 °C, 200 r/min), the tubes were centrifuged for 15 min (1850× g), and the supernatants were analyzed for the target pollutants.

### 2.4. Chemical Analysis

#### 2.4.1. Analysis of Pyrene

For each tube, 10 mL of the collected supernatant was extracted three times with 30 mL of n-hexane (10 mL each time); the collected organic liquid was concentrated at 1 mL via rotary evaporation (N-1100, EYELA, Tokyo, Japan), and the 1 mL liquid was transferred to a 1.5 mL vial for analysis. Phe-d8 and pyr-d10 were used as recovery indicator and internal standard, respectively. A Gas Chromatography Mass Spectrometry with an electron ionization source (6890/5973N, Agilent, Poway, CA, USA) and a DB-5ms capillary column (30 m × 250 μm × 0.25 μm) (Agilent, Santa Clara, CA, USA) were used for the measurement. Helium gas with a flow rate of 2 mL/min was used as the carrier gas, and the inlet temperature was 280 °C. The column temperature program was set as follows: maintained at 50 °C for 1 min, raised the temperature to 200 °C at 15 °C/min, raised the temperature to 305 °C at 20 °C/min, and maintained at 305 °C for 3 min. The ion source temperature was 245 °C, and the fourth stage rod temperature was 150 °C. The concentration of pyrene standards ranged from 1 μg/L to 100 μg/L. The instrument detection limit of pyrene was 0.70 μg/L. A standard solution of pyrene (100 μg/L) was measured every 20 samples to control the deviation of instrument performance, and the obtained deviation was within 20%.

#### 2.4.2. Analysis of As

For each tube, 1 mL of the collected supernatant passed through a 0.22 μm filter membrane, was diluted with ultrapure water to 10 mL, and then was analyzed using a Liquid Chromatography Atomic Fluorescence Spectrometer (LC-AFS, LC-AFS6500, Haiguang, Beijing, China) with an anion exchange column (Hamilton PRP-X100, 250 mm × 4.1 mm × 10 μm) and hollow cathode lamps. Phosphate buffer was used as the mobile phase for the liquid chromatography (LC) system, with a flow rate of 1 mL/min. A 10% HCl solution and 3.5% KBH_4_ solution were respectively used as the carrier and reducing agent for the atomic fluorescence spectroscopy (AFS) system, and the main current and auxiliary current were 300 mA and 150 mA, respectively. At a flow rate of 300 mL/min and shield flow rate of 900 mL/min, and under negative high pressure of 300 V, the ionic arsenic was reduced to atomic arsenic, with different species of As being separated. The concentrations of As (III) and As (V) standards both ranged from 1 μg/L to 200 μg/L. The detection limits for As (III) and As (V) were 0.545 μg/L and 0.837 μg/L, respectively. A standard solution of As (III) and As (V) (100 μg/L for each species) was measured every 20 samples to control the deviation of instrument performance, and the obtained deviations were within 10%.

### 2.5. Data Analysis

The adsorption processes of pyrene and As (III) using micro/nano carbon black and iron oxide were simulated by quasi-first-order and quasi-second-order kinetic models, as shown in Equations (1) and (2).

(1) $$\ln\left(q_{e}-q_{t}\right)=lnq_{e}-k_{1}t$$

(2) $$\frac{t}{q_{t}}=\frac{1}{k_{2}q_{e}^{2}}+\frac{1}{q_{e}}t$$

where *t* is the adsorption time (h), *q* and *q_t_* are the equilibrium adsorption capacity and adsorption capacity at time *t* (μg/g or mg/g), and *k*_1_ and *k*_2_ are the adsorption rates of pseudo-first-order kinetics (h^−1^) and pseudo-second-order kinetics (mg(g·h)^−1^ or μg(g·h)^−1^), respectively.

Three adsorption/desorption isotherm models were used to describe the equilibrium process of pollutants between solid and liquid phases, as shown in Equations (3)–(5).

(3) $$q_{e}=K_{H}C_{e}$$

(4) $$\frac{1}{q_{e}}=\frac{1}{q_{max}K_{L}}\frac{1}{Ce}+\frac{1}{q_{max}}$$

(5) $$lgq_{e}=\frac{1}{n}lgCe+lgK_{F}$$

where *q_e_* and *q_max_* are the equilibrium adsorption capacity and maximum adsorption capacity (μg/g or mg/g), *C_e_* is the equilibrium concentration in the liquid (μg/L), *K_H_* is adsorption coefficient for the Henry model (L/g), *K_L_* is a constant for the Langmuir model (L/mg), *K_F_* (L/mg) and *n* (g/L) are constants for the Freundlich model. 

The model fitting and data visualization were performed using Origin2022. Data between treatments and controls were compared by *t*-test using SPSS 26.0, with a significance level set at 0.05. Other statistical analyses of the data were conducted using EXCEL 2016.

## 3. Results

### 3.1. Characterizations of Micro/Nano Carbon Black and Iron Oxide

**Figure 1:**
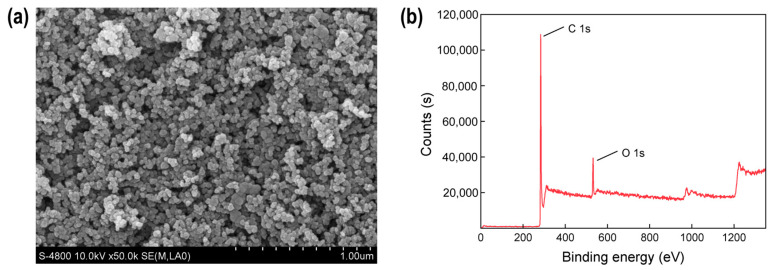
Characterization diagram of micro/nano carbon black. (**a**) SEM; (**b**) XPS.

The characterizations of micro/nano carbon black are shown in Figure 1. The SEM image shows that the microstructure of micro/nano carbon black is spherical-like, with irregular protrusions on the surface and relatively uniform sizes, with a diameter of 100–200 nm. The XPS spectrum shows that there are high-intensity peaks in the binding energies of the C1s and O1s orbitals on the binding energy spectrum of the sample, while there are low-intensity peaks in the binding energies of the S2p and N1s orbitals. According to the quantitative results, the proportion of each element’s content (in atomic number, except for H) in the carbon black is C (94.03%), O (4.49%), N (1.11%), and S (0.37%).

**Figure 2:**
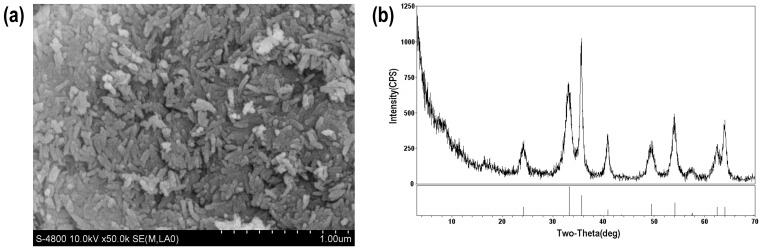
Characterization diagram of micro/nano iron oxide. (**a**) SEM; (**b**) XRD.

The characterizations of micro/nano iron oxide are shown in Figure 2. The SEM image shows that the micro/nano iron oxide has an irregular rod-shaped structure with significant size differences. Most individual rods are about 1 µm long and 100–200 nm wide. The XRD pattern shows that there are six typical diffraction peaks that appeared in the 2θ diffraction angle of iron oxide, which are consistent with the diffraction peaks of hematite in the standard database, indicating that the iron oxide is hematite.

### 3.2. Adsorption Kinetics of Pyrene and As (III) by Micro/Nano Carbon Black and Iron Oxide

The pre-experiments showed that at a micro/nano carbon black dosage of less than 100 mg in 40 mL water containing 10 mg/L As (III), the micro/nano carbon black had little adsorption of As (III); however, when the dosage of micro/nano carbon black was larger than 100 mg, As (III) was oxidized to As (V). At a dosage of micro/nano iron oxide ranging from 0–1000 mg in 40 mL water containing 80 μg/L pyrene, the micro/nano iron oxide had little adsorption of pyrene (Figure S1). 

**Figure 3:**
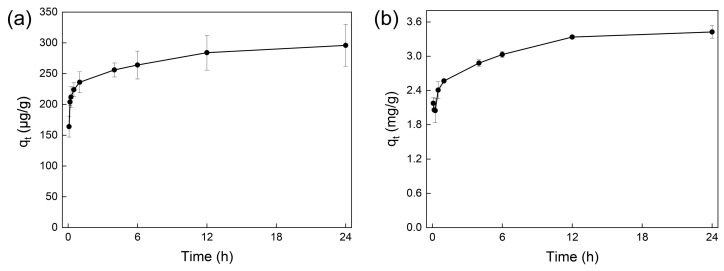
The adsorption kinetics of pyrene and As (III) by micro/nano carbon black and iron oxide. (**a**) Adsorption kinetics of pyrene by micro/nano carbon black. (**b**) Adsorption kinetics of As (III) by micro/nano iron oxide.

Adsorption kinetics were studied for the adsorption of pyrene using micro/nano carbon black and the adsorption of As (III) using micro/nano iron oxide at optimal dosages of sorbents (5 mg for carbon black and 10 mg for iron oxide). The adsorption kinetics data are shown in Figure 3. Both adsorptions reached equilibrium within 24 h, and the adsorption data are fit well by both quasi-first-order and quasi-second-order kinetic models, with the latter being better (Table 2). This indicated that the mechanisms of both adsorptions were mainly chemical reactions. At equilibrium (24 h), the maximum adsorption capacity of micro/nano carbon black for pyrene was 283.23 μg/g, while the maximum adsorption capacity of micro/nano iron oxide for As was 3.45 mg/g.

### 3.3. Adsorption Isotherms of Pyrene and As (III) by Micro/Nano Carbon Black and Iron Oxide

**Figure 4:**
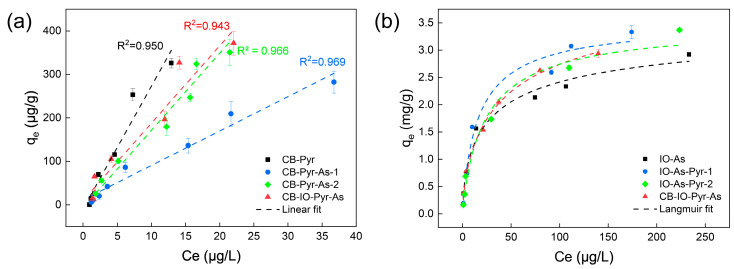
Adsorption isotherms and fitting model parameters of micro/nano particles for pyrene and As (III) (Details on the adsorption systems are shown in Table 1). (**a**) Adsorption isotherms of pyrene on micro/nano carbon black, The black, blue, green and red lines represent the linear fit for CB-Pyr, CB-Pyr-As-1, CB-Pyr-As-2 and CB-IO-Pyr-As, respectively; (**b**) Adsorption isotherms of As (III) by micro/nano iron oxide, The black, blue, green and red lines represent the linear fit for IO-As, IO-As-Pyr-1, IO-As-Pyr-2 and CB-IO-Pyr-As, respectively. The colors of the fit lines are corresponding to the colors of the legends.

The adsorption isotherms of pyrene using micro/nano carbon black are shown in Figure 4a. Under the study conditions, the adsorption capacity of micro/nano carbon black for pyrene increased with the initial concentration of pyrene in the liquid, and the equilibrium process could be a good fit for the linear Henry model. The k-values in the different systems had the following order: CB-Pyr (28.171 L/g) > CB-IO-Pry-As (17.785 L/g) > CB-Pyr-As-2 (16.581 L/g) > CB-Pyr-As-1 (7.914 L/g), as shown in Table 3. The presence of As (III) reduced the adsorption coefficient of the micro/nano carbon black for pyrene, and this impact increased with an increased concentration of As (III). The presence of iron oxide slightly reduced the impact of As (III) due to its adsorption of As (III). Even though the iron oxide could adsorb As (III), the adsorption coefficient in the CB-IO-Pry-As system was much lower than that in the CB-Pyr system.

The adsorption isotherms of As (III) using micro/nano iron oxide are shown in Figure 4b. Under the study conditions, the adsorption capacity of the micro/nano iron oxide for As (III) increased with the initial concentration of As (III) in the liquid, and the equilibrium process could be a good fit for the Langmuir model and Freundlich model, with the former being better (Table 3). The q_e_ values in the different systems had the following order: IO-As (2.762 mg/g) < CB-IO-Pry-As (3.366 mg/g) < IO-As-Pyr-2 (3.367 mg/g) < IO-As-Pyr-1 (3.827 mg/g), as shown in Table 3. The presence of pyrene increased the adsorption capacity of the micro/nano iron oxide for As (III), and this impact increased with an increased concentration of pyrene. The presence of carbon black barely reduced the impact of pyrene, even though the carbon black easily adsorbed the pyrene. The adsorption capacity in the CB-IO-Pry-As system was much higher than in the IO-As system. The obtained q_e_ values of As (III) were comparable with those reported in the literature. However, the obtained q_e_ values of pyrene were much less than those reported in the literature, and the reason was that the initial concentrations in our study were much less than those in the reported studies (Table S2).

The authors understand that the adsorption capacity of a material increases with an increasing adsorbate concentration; therefore, a comparative table must be prepared with other adsorbents present in the literature for the same purpose, describing the maximum capacity obtained and the concentration used in each study.

### 3.4. Desorption Isotherms of Pyrene and As (III) from Micro/Nano Carbon Black and Iron Oxide

**Figure 5:**
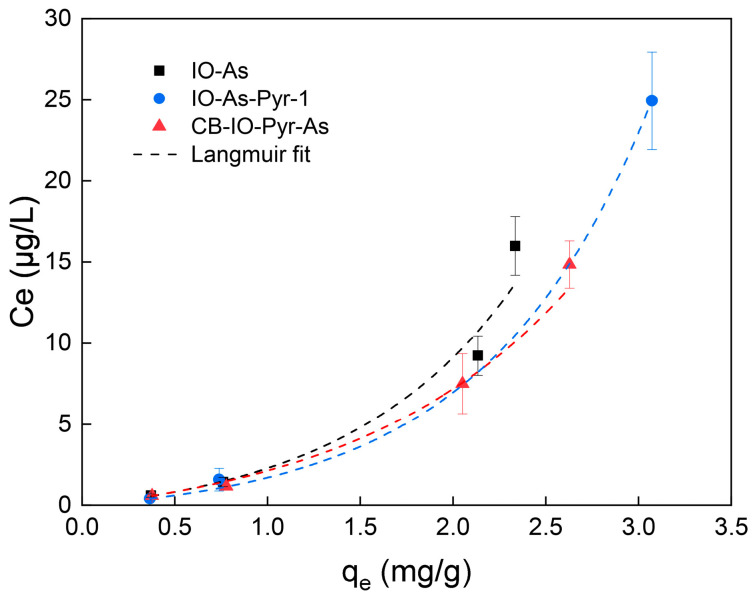
Desorption isotherms on micro/nano iron oxide, The black, blue and red lines represent the linear fit for IO-As, IO-As-Pyr-1 and CB-IO-Pyr-As, respectively (Details on the adsorption systems are shown in Table 1). The colors of the fit lines are corresponding to the colors of the legends.

In the desorption experiment, the desorption of pyrene was not detected in any of the studied systems, but a slight desorption of As (III) was detected. Figure 5 shows the desorption isotherms of As (III) in the IO-As, CB-IO-Pry-As, and IO-As-Pyr-1 systems. The desorption capacity of As (III) increased with the initial loading of As (III) in the solid phase, and the equilibrium process could be described by both the Langmuir model and the Freundlich Model, with the former being better, as shown in Table 4. Due to residual As (III) in the liquid at the beginning of the desorption experiment, there were certain errors in the calculation of the desorption isotherm parameters. Nevertheless, it could be seen that the presence of pyrene reduced the desorption of As (III). Overall, the desorption rates of As (III) were small (less than 4%).

## 4. Discussion

### 4.1. Adsorption Mechanism

This study showed that micro/nano carbon black had a strong adsorption of the pyrene but hardly adsorbed As (III), while micro/nano iron oxide had a strong adsorption of As (III) but hardly adsorbed the pyrene. 

Previous studies have shown that the adsorption mechanisms of carbon materials on PAHs included hydrophobic interactions, van der Waals forces, and π bond interactions [12,20,21]. Micro/nano carbon black has a large specific surface area, and its surface hydrophobicity could create favorable conditions for the adsorption of pyrene [22]. In the production of micro/nano carbon black, high-temperature reactions of carbon-containing substances might form some aromatic functional groups, which play an important role in the adsorption of pyrene [23]. In addition to π–π interactions, N and O atoms in carbon-based materials could provide lone-pair electrons and empty orbitals, forming N–P interactions with π electrons in PAHs. At the same time, some polar functional groups on carbon adsorbents can also enhance their polarity, which enhances the adsorption of PAHs by using carbon materials in dipole interactions [13,22]. In this study, we attempted to characterize the functional groups on the surface of micro/nano carbon black using the FITR method, but due to the high blackness of the carbon material and its overall strong absorption of infrared radiation, the functional groups could not be identified. However, an XPS analysis detected certain amounts of O, N, and S, which might form polar hydrophobic functional groups. After being adsorbed by the micro/nano carbon black, the pyrene was difficult to desorb, indicating that the adsorption reaction was very strong.

**Figure 6:**
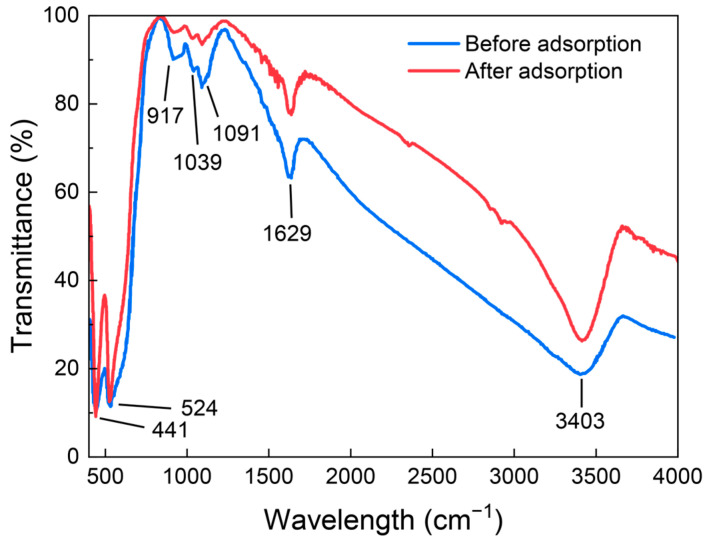
Changes in the FTIR spectra of micro/nano iron oxide before and after the adsorption of As (III).

Micro/nano iron oxide had strong adsorption of As (III), which was related to its large specific surface area and abundant hydroxyl functional groups on the surface. In aqueous solutions, the surfaces of metal oxides were covered by many hydroxyl groups, which originated from the metal oxides themselves or the dissociation of water molecules [14]. The surface functional groups of micro/nano iron oxide were mainly composed of bound water and –OH. After adsorbing As (III), the –OH absorption peaks at the wave numbers of 1091 cm^−1^, 1039 cm^−1^, and 917 cm^−1^ were significantly weakened, indicating that surface hydroxyl groups participated in the adsorption process of As (III) using micro/nano iron oxide (Figure 6). The hydroxyl groups on the surface of the iron oxide were able to form complexes with various heavy metal ions to achieve adsorption, among which arsenite was a good hydroxyl ligand [15]. The adsorption process first formed physical adsorption through hydrogen bonding between O of the arsenite and H of the hydroxyl group. The arsenite physically adsorbed on the surface of the iron oxide was further transformed into monodentate or bidentate complexes. The Gibbs activation free energy of this process was often low (less than 0), making the reaction easy to occur spontaneously [24]. Once they exchanged with the hydroxyl groups on the surface of the iron oxide through a ligand exchange to form inner spherical complexes, it was difficult to detach them from the surface of the iron oxide.

### 4.2. The Influence of Co-Existing Pollutants on the Adsorption 

Although the micro/nano carbon black did not adsorb As (III), the presence of As (III) reduced the adsorption of the pyrene when using micro/nano carbon black. The addition of micro/nano iron oxide slightly reduced the impact of As (III) on the adsorption of pyrene when using micro/nano carbon black. On the other hand, although the micro/nano iron oxide did not adsorb the pyrene, the presence of the pyrene increased the adsorption of As (III) when using the micro/nano iron oxide. The addition of the micro/nano carbon black did not reduce the impact of the pyrene on the adsorption of As (III) when using micro/nano iron oxide. 

As (III) reduced the adsorption of pyrene when using micro/nano carbon black, which might be related to the pH change in the solution after the addition of arsenite. This study showed that arsenite is a weak acid that could bind with protons and hydrolyze in an aqueous solution, and it mainly exists in the forms of H_3_AsO_3_ and H_2_AsO_3_^−^, increasing the pH of the aqueous solution [25]. A higher pH was not conducive to the adsorption of the pyrene when using micro/nano carbon black as OH- in the solution bound to the surface of the carbon black. The negative charge on the surface of the micro/nano carbon black increased the electrostatic resistance that the electron-rich pyrene needed to overcome when being adsorbed on the surface, thereby reducing the adsorption efficiency [26]. Other studies have suggested that in an aqueous solution of pyrene, which is different from a small volume of metal cations (such as Li^+^), which are preferentially bound to water molecules to form stable hydration shells, larger volume metal cations (such as K^+^) are more likely to preferentially bind to the pyrene through cation−π bonding, which might compete for the π bonding sites of the pyrene with micro/nano carbon black [27]. In a study by Eeshwarasinghe et al., heavy metals such as Cd, Cu, and Zn reduced the adsorption capacities of PAHs by activating carbon particles, which was related to the decrease in negative zeta potential [28]. 

Pyrene could increase the adsorption of As (III) when using micro/nano iron oxide, which might be due to the strong affinity of pyrene with protons dissociated from water, thereby promoting water ionization and producing more OH–. These OH– are further combined with micro/nano iron oxide to form hydroxyl radicals on its surface, providing more adsorption sites for arsenite [29,30]. Due to the large electron-rich conjugated π bond in aromatic compounds as well as the electron-deficient state of H and protons in water molecules, pyrene molecules might combine with protons to form O-H ····π or H+····π forces, which are electrostatic forces similar to hydrogen bonds and promote water ionization [31]. So far, numerous theoretical calculations and experimental analyses using benzene as a model have confirmed that the molecular process of Benzenem-H_2_On-H+ (m represents the number of benzene molecules) is widely present in aqueous benzene solutions [32,33]. This may provide new approaches for the remediation of environments when there is combined pollution of PAHs and As. In a study by Zhang et al., there was a synergistic effect on the adsorptions of pyrene and Cu (II) when using silica adsorbents doped with Fe (III), which might be attributed to the formation of pyrene-Cu (II) complexes [34].

We should be careful when dealing with nanomaterials to remediate the environment. Nanomaterials may cause toxicity to microbes and multi-cellular organisms [35]. In addition, the widespread application of nanomaterials in remediation has been hampered by challenges in accurately delivering them to contaminated sites due to their rapid aggregation and/or retention. An understanding of the processes that influence the environmental transportation and fate of nanomaterials is critical for optimizing environmental applications and assessing risks [36].

## 5. Conclusions

This study investigated the adsorption of pyrene and As (III) when using micro/nano carbon black and iron oxide under individual and co-existing conditions. The micro/nano carbon black easily adsorbed the pyrene (pseudo-second-order rate constant of 0.016 mg/(g·h) and adsorption capacity of 283.23 μg/g at 24 h), while the micro/nano iron oxide easily adsorbed As (III) (pseudo-second-order rate constant of 0.814 mg/(g·h) and adsorption capacity of 3.45 mg/g at 24 h). The mechanisms of the adsorptions were mainly chemical reactions. The adsorption isotherms of the pyrene best fit the Henry model, while the adsorption isotherms of As (III) best fit the Langmuir model. The presence of As (III) reduced the adsorption of the pyrene when using micro/nano black carbon, and this impact increased with an increase in the As (III) concentration. The presence of pyrene increased the adsorption of As (III) when using micro/nano iron oxide, and this impact increased with an increase in the pyrene concentration. The interactions between the micro/nano carbon black and micro/nano iron oxide were small. These results provide guidance for the risk management and remediation of the environment when there is a combined pollution of PAHs and As.

There were limitations in this study. The functional groups on the surface of the micro/nano carbon black could not be measured using the FITR method, and, therefore, the deeper reaction mechanism was not clarified. In addition, factors influencing the adsorptions of the pyrene and As (III) when using the micro/nano carbon black and iron oxide were not investigated. In future studies, the mechanisms and influencing factors of the impact of the co-existence of pyrene and As (III) on their adsorptions should be explored. 

## Figures and Tables

**Table 1:** Setup of the experiments of adsorption isotherms.

System Label	Micro/Nano Iron Oxide (mg)	Micro/Nano Carbon Black (mg)	Pyrene ^a^ (µg/L)	As (III) ^b^ (mg/L)
IO-As	10	0	0	0.01–1
IO-As-Pyr-1	10	0	80	0.01–1
IO-As-Pyr-2	10	0	1–80	0.01–1
CB-Pyr	0	5	1–80	0
CB-Pyr-As-1	0	5	1–80	1
CB-Pyr-As-2	0	5	1–80	0.01–1
CB-IO-Pyr-As	10	5	1–80	0.01–1

^a^ 1–80 refers to initial concentration of pyrene at 1, 2, 5, 10, 20, 40, 60, 80 µg/L; ^b^ 0.01–1 refers to initial concentration of As (III) at 0.01, 0.02, 0.05, 0.1, 0.2, 0.4, 0.6, 0.8, 1 mg/L.

**Table 2:** The values of parameters in the adsorption kinetic models.

**System**	**Pseudo-first-order kinetics**			**Pseudo-second-order kinetics**		
	**q_e_ (mg/g)**	**k_1_ (/** **h** **)**	**R^2^**	**q_e_ (mg/g)**	**k_2_ (** **mg/(g·h)** **)**	**R^2^**
IO-As	3.425	0.217	0.982	3.446	0.814	0.998
						
**System**	**Pseudo-first-order kinetics**			**Pseudo-second-order kinetics**		
	**q_e_ (μg/g)**	**k_1_(/** **h** **)**	**R^2^**	**q_e_ (μg/g)**	**k_2_ (** **μg/(g·h))**	**R^2^**
CB-Pyr	296.000	0.172	0.936	283.225	0.016	0.998

**Table 3:** The values of parameters in the adsorption isotherms models.

System *	Langmuir Model			Freundlich Model		
	K_L_ (L/mg)	q_e_ (mg/g)	R^2^	K_F_ (L/mg)	*n* (g/L)	R^2^
IO-As	0.117	2.760	0.951	0.333	2.298	0.930
IO-As-Pyr-1	0.060	3.827	0.986	0.294	1.984	0.935
IO-As-Pyr-2	0.059	3.367	0.987	0.250	1.946	0.947
CB-IO-Pyr-As	0.079	3.366	0.954	0.294	1.967	0.951
	
System *	**Henry Model**					
	**K_H_ (** **L/g** **)**			**R^2^**		
CB-Pyr	28.171			0.950		
CB-Pyr-As-1	7.914			0.969		
CB-Pyr-As-2	16.581			0.966		
CB-IO-Pyr-As	17.785			0.943		

* In IO-As-Pyr-1, the concentrations of As (III) ranged from 0.01 to 1 mg/L, and the concentration of pyrene was kept at 80 μg/L; in IO-As-Pyr-2, the concentrations of As (III) ranged from 0.01 to 1 mg/L, and the concentration of pyrene ranged from 1 to 80 μg/L; in CB-Pyr-As-1, the concentrations of pyrene ranged from 1 to 80 μg/L, and the concentration of As (III) was kept at 1 mg/L; in CB-Pyr-As-2, the concentration of pyrene ranged from 1 to 80 μg/L, and the concentration of As (III) ranged from 0.01 to 1 mg/L.

**Table 4:** Langmuir and Freundlich models for desorption isotherms.

System	Langmuir Model			Freundlich Model		
	K_L_ (L/mg)	q_e_ (mg/g)	R^2^	K_F_ (L/mg)	*n* (g/L)	R^2^
IO-As	0.222	3.103	0.999	0.546	1.764	0.978
IO-As-Pyr-1	0.400	2.532	0.959	0.628	1.796	0.946
CB-IO-Pyr-As	0.181	3.946	0.990	0.592	1.709	0.972

## Data Availability

The data presented in this study are available on request from the corresponding author. The data are not publicly available due to privacy.

## Supplementary Materials

The following supporting information can be downloaded at https://www.mdpi.com/article/10.3390/toxics12040251/s1, Figure S1: Adsorption of pyrene and As (III) by micro/nano carbon black and iron oxide; Table S1: Setup of Pre-Experiment; Table S2: Adsorption isotherms of As (III) and pyrene reported in the literature [37,38,39,40,41,42].
