# Supplementary material for: Adsorption of Pyrene and Arsenite by Micro/Nano Carbon Black and Iron Oxide
Source: Toxics. 2024 Mar 29;12(4):251. doi: 10.3390/toxics12040251 (PMC11053581; doi:10.3390/toxics12040251)
Supplement: Supplementary file 1 [file toxics-12-00251-s001.zip › toxics-2904458-supplementary.pdf]

# Supplementary Materials

Table S1. Setup of Pre-Experiment.

| System Labels | Micro/nano hematite a (mg) | Micro/nano carbon black a (mg) | Pyrene (μg/L) | As (III) (mg/L) |
|---|---|---|---|---|
| IO-As | 0-1000 | 0 | 0 | 10 |
| IO-Pyr | 0-1000 | 0 | 80 | 0 |
| CB-Pyr | 0 | 0-1000 | 80 | 0 |
| CB-As | 0 | 0-1000 | 0 | 10 |

a 0-1000 refers to dosages of micro/nano hematite and carbon black ranged from 0 to 1000 mg (0, 5, 10, 25, 50, 100, 500, and 1000 mg)

Table S2. Adsorption isotherms of As(III) and pyrene reported in the literatures

| Adsorbent | Adsorbate | Initial concentration | Isotherm model | qmax (mg/g) | References |
|---|---|---|---|---|---|
| Iron oxide nanoparticles | As(III) | 1–7 mg/L | Langmuir | 2.9 mg/g | [37] |
| Magnetite particles | As(III) | 2 mg/L | Langmuir | 3.70 mg/g | [38] |
| $Fe_3O_4$ nanoparticles | As(III) | 32.32 mg/L | Langmuir | 7.18 mg/g | [39] |
| Mixed $\alpha$- $Fe_2O_3$ and$\gamma$-$Fe_2O_3$ | As(III) | 0–60 mg/L | Langmuir | 46.5 mg/g | [30] |
| Carbon Nanotubes | pyrene | 15 mg/L | Dubinin-Ashtakhov | 42.7 mg/g | [40] |
| Activated Carbon | pyrene | 8 mg/ | Langmuir | 104.5 mg/g | [41] |
| regenerable graphene wool | pyrene | 300-800 ng/L | Langmuir | 20 mg/g | [42] |

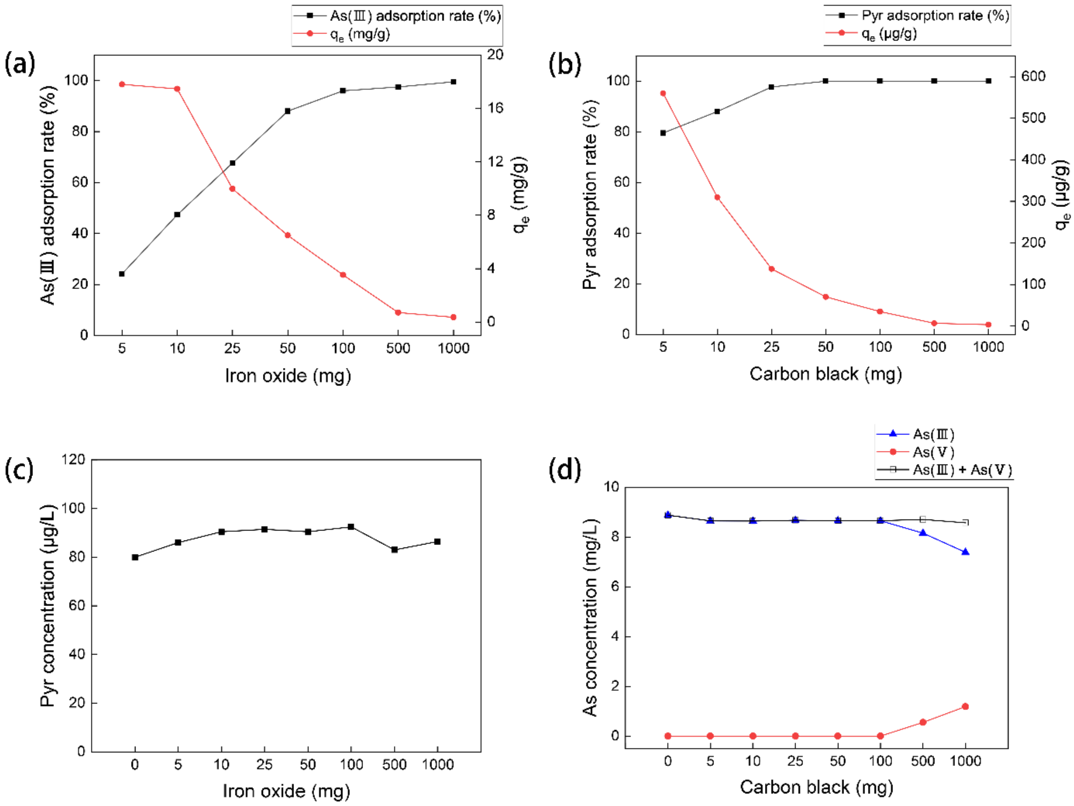


Figure S1. Adsorption of pyrene and As (III) by micro/nano carbon black and iron oxide.

(a) Adsorption rate and adsorption capacity of As (III) over the dosage of micro/nano iron oxide;

(b) Adsorption rate and adsorption capacity of pyrene over the dosage of micro/nano carbon black;

(c) Concentration of pyrene in the liquid over the dosage of micro/nano iron oxide;

(d) Concentration of f As in the liquid over the dosage of micro/nano carbon black.
